# Pediatric Obstructive Sleep Apnea: Knowledge and Attitudes of Medical and Dental Students and Fresh Graduates from Saudi Arabia

**DOI:** 10.3390/children8090768

**Published:** 2021-08-31

**Authors:** Lamyaa N. Alharbi, Mashail A. Alsaikhan, Sanaa N. Al-Haj Ali, Ra’fat I. Farah

**Affiliations:** 1 College of Dentistry, Qassim University, Qassim 51452, Saudi Arabia; Lamya.alharbi@qudent.org (L.N.A.); Mashail.alsaikhan@qudent.org (M.A.A.); 2 Department of Orthodontic and Pediatric Dentistry, College of Dentistry, Qassim University, Qassim 51452, Saudi Arabia; 3 Department of Prosthetic Dental Sciences, College of Dentistry, Qassim University, Qassim 51452, Saudi Arabia; dr.rafat.farah@qudent.org

**Keywords:** dental students, education, graduates, medical students, pediatric obstructive sleep apnea

## Abstract

This study aimed to assess the knowledge level and attitudes of graduating Saudi medical and dental students and fresh graduates from those faculties about pediatric obstructive sleep apnea (OSA), and the relation of their knowledge level to sociodemographic variables. In this cross-sectional study, 722 graduating students and fresh graduates were requested to answer a questionnaire pretested for validity and reliability. The data were analyzed statistically. Results revealed that medical participants scored 15.45 (out of 22), with 38% of them showing good knowledge about pediatric OSA, while dental participants scored 14.59, with 25.2% of them showing good knowledge. By regression analysis, medical participants (odds ratio (OR): 1.529) were more likely to have good knowledge than dental participants, while participants who belonged to institutions located in the central region (OR: 0.546) were less likely to have good knowledge than those from southern region institutions. In addition, participants from public institutions (OR: 0.290) were less likely to have good knowledge than those from private institutions. Regarding attitudes, medical participants scored 14.13 (out of 20), and dental participants scored 14.64. We detected a significant positive correlation between knowledge and attitude scores of dental participants. Given these findings, the knowledge level of graduating Saudi medical and dental students and fresh graduates about pediatric OSA was not optimal. The college type, institution sector, and location in the kingdom were factors associated with good knowledge. There is a need for further education and training about pediatric OSA in the undergraduate Saudi medical and dental curricula and continuing professional development programs about the topic after graduation.

## 1. Introduction

According to the American Academy of Sleep Medicine, obstructive sleep apnea (OSA) is one of the most common sleep disorders but is often underdiagnosed and untreated [1]. It is defined as repeated episodes of upper airway closure during sleep, resulting in oxyhemoglobin desaturation, and episodes of apnea or hypopnea in a sleep recording that cause sleep fragmentation [2,3]. The frequency of these episodes determines the severity of the condition [3]. Other than respiratory disease, OSA has been associated with metabolic, cardiovascular, dental, and other diseases. In children, undiagnosed and/or untreated OSA can also be associated with impaired growth and learning and behavioral problems, with consequent poor school performance [4,5].

Pediatric OSA differs from adult OSA since it exhibits characteristics that are specific to growing subjects [4]. In childhood OSA, there is no clear correlation between the severity of the clinical presentation and daytime symptoms, with a more nuanced range of symptoms [6].

If a child is suspected of being at risk for OSA, a referral to the appropriate medical specialist (e.g., otolaryngologist, pulmonologist, sleep medicine physician) is advised [7], where the condition can be confirmed by overnight laboratory-based polysomnography [8]. Consequently, physicians’ knowledge should be the key factor in eliciting a history of pediatric OSA and making referrals for OSA evaluation [9]. Dentists are also involved in the same process, as they frequently encounter children at an early age in their practice [10], and their attention is focused on the orofacial sector. Consequently, their role in screening children for OSA and referring children at risk for the appropriate medical provider has been strongly emphasized [4,7,11,12,13]. Proper knowledge of physicians and dentists about pediatric OSA should ensure better cooperation and consequently early detection and adequate treatment planning for affected children [14], to prevent morbidity and sequela and to provide a better quality of life for both the child and his or her family members [15]. Ensuring adequate undergraduate education would be a good starting point [16,17], particularly after considering the deficiencies in the knowledge of dentists [14,17,18,19] (as well as physicians and fresh medical graduates) about OSA that have been reported in Saudi Arabia as well as elsewhere [2,9,15,20,21,22,23,24,25]. Furthermore, comparative studies of knowledge of medical and dental students or even graduates about pediatric OSA are lacking; therefore, this study aimed to assess the knowledge level and attitudes of graduating medical and dental students as well as fresh graduates from Saudi Arabia about pediatric OSA and the relation of their knowledge level to sociodemographic variables. The tested null hypotheses were that (1) there is no difference in the knowledge and attitudes about pediatric OSA between participants from both fields, and (2) the sociodemographic variables have no impact on the knowledge of the participants.

## 2. Methods

### 2.1. Study Population and Ethical Approval

This was a cross-sectional study conducted on a convenience sample of 722 graduating medical and dental students and fresh graduates (interns). The participants were approached personally as well as through social networking sites due to the coronavirus disease 2019 (COVID-19 pandemic) which prohibited direct communication with all participants, given the e-learning educational system which was dominant in the study period (December 2020–April 2021). All participants in the current study provided their written consent. Ethical approval was also obtained from the ethical committee of the College of Dentistry, Qassim University before the start of the study (reference number: EA/6060/2020, issued on 26 December 2020). The inclusion criteria for the study were graduating medical and dental students from any Saudi institution, as well as fresh graduates (interns only) of those faculties; participants who provided their written consent to participate in the study; and those who had an account on social networking sites for those who were not contacted in person.

### 2.2. Study Measures

The participants were either handed or sent a structured electronic questionnaire in the English language during the period from December 2020 through April 2021. The questionnaire was written by a panel of three specialists and subjected to face validity. A few of the questions (a limited proportion) were brought from the Obstructive Sleep Apnea Knowledge and Attitudes in Children (OSAKA-KIDS) questionnaire [26]. The rest of the questions were written according to the latest policy about OSA released by the American Academy of Pediatric Dentistry [7]. The questionnaire consisted of 33 items, was in three parts, and aimed to assess (1) the sociodemographic profile of participants and whether their curriculum included information about pediatric OSA; (2) participants’ general knowledge about pediatric OSA including its epidemiology, diagnosis, risk factors, signs and symptoms, general findings, dental treatment need, and referral (the response options to these questions were “true”, “false”, or “do not know”); (3) participants’ attitude concerning the importance of OSA as a clinical disorder in children and the importance of identifying children with this disorder, their self-assessed confidence in diagnosing children at risk for OSA, and their interest in further education about the topic. Response options to these questions were scored on a 5-point Likert scale, ranging from 1 (not important) to 5 (extremely important), and from 1 (strongly disagree) to 5 (strongly agree).

To ensure content validity, the questionnaire was piloted among a sample of 10 participants to ensure that all questions were clear and necessary modifications were performed. The pilot sample was then excluded from the study’s main sample. Additionally, reliability testing of the questionnaire was conducted via a test–retest format in which all questions scored a correlation level above 0.8. 

### 2.3. Data Analysis 

Statistical analysis of the data was conducted using the SPSS software (version 22.0; IBM Corporation, Armonk, NY, USA (for Windows^®^)). Simple frequency distributions and percentages of the participants’ responses toward the sociodemographic questions (part 1) and attitude questions (part 3) were produced. Additionally, frequency distributions and percentages of the participants’ correct responses in the questions which assessed participants’ knowledge of pediatric OSA (part 2) were produced. Frequency responses of part 2 questions were compared using the chi-square test (univariate approach). Participants were given a knowledge score that ranged between 0 and 22 based on their answers to that part. Furthermore, they were given an overall attitude score based on their answers to part 3 questions which ranged from 4 to 20. The overall knowledge and attitude scores of the participants were expressed as mean ± SD and compared according to the college type. Then, participants were classified into one of two categories according to their knowledge score; those with good knowledge about pediatric OSA (scored ≥16 out of 22 or correctly answered 70% or more of part two questions) and those with poor knowledge (scored <16 out of 22 or answered correctly <16 out of 22 questions). A chi-square test was then used to detect the sociodemographic variables which were associated with good knowledge of the participants. Binary logistic regression (multivariate approach) was further used to ascertain the sociodemographic variables which were associated with having good knowledge. Only variables that showed an association in the univariate approach were entered in the regression model. The relationship between the knowledge and attitude scores of the participants was also determined using Spearman’s correlation coefficient. Probability values of *p* < 0.05 were considered statistically significant throughout.

## 3. Results

The questionnaires were completed and returned by 722 (all) participants, of whom 41.1% were from the medical field, while 58.9% were from the dental field. The mean knowledge score of participants was 14.95 ± 2.89 out of 22, that of medical participants was 15.45 ± 2.63, and dental participants scored 14.59 ± 3.01. The mean attitude score of medical participants was 14.13 ± 1.90 out of 20, compared to 14.64 ± 1.89 for dental participants.

Table 1 and Table 2 show the participants’ correct responses for each question, which assessed their knowledge about pediatric OSA and its associated signs and symptoms. The percentage of participants who showed good knowledge about pediatric OSA was 30.5%. Around 38.0% of participants from the medical field had good knowledge about pediatric OSA compared to 25.2% of participants from the dental field. Ten questions assessed the participants’ general knowledge of pediatric OSA, and twelve questions assessed their knowledge of its associated signs and symptoms. Out of ten general knowledge questions, four questions were answered correctly by >70% of the participants from both fields. These questions primarily focused on the diagnosis of pediatric OSA, its risk factors, and the referral of suspected cases. A greater proportion of medical participants knew that intra-oral appliances can be required in the treatment of pediatric OSA (67.7% vs. 46.1%) and that children with OSA can have learning deficits (41.1% vs. 24.3%) compared to dental participants, while the opposite was the case in the question about congestive heart failure as a cause for pediatric OSA (37% vs. 56.2%, respectively). Less than a third of the participants from both fields knew the correct answer in the questions which concerned the epidemiology of the condition, and the possibility of having attention deficits or hyperactivity in affected children. Statistically significant differences existed between participants from both fields with regards to their answers in five of the questions in this section (*p* < 0.05) (Table 1). 

With regards to knowledge of participants about signs and symptoms of pediatric OSA, better knowledge of participants from both fields was found (Table 2). The great majority of the participants from both fields identified correctly nine out of twelve reported signs and symptoms as associated with pediatric OSA. Around 56% of medical participants and 44% of dental participants knew that attention problems are considered among the signs and symptoms, while less than a third of them knew that sweating and bedwetting are associated with pediatric OSA. Statistically significant differences existed between participants from both fields with regards to the identification of eight signs and symptoms (*p* < 0.05) (Table 2).

Table 3 shows the sociodemographic background of the participants, the percentage of participants who had good knowledge according to each sociodemographic variable, and the sociodemographic variables which were associated with good knowledge of the participants according to the chi-square test and binary logistic regression analysis. According to the chi-square test, four variables were found to be associated with good knowledge of the participants; these included the participant’s college, the geographic location of the participant’s institution in the kingdom, the sector of the participant’s institution, and curriculum inclusion of information about pediatric OSA (*p* < 0.05). Of these variables, the first three prove to have a statistically significant effect on the good knowledge of the participants according to logistic regression analysis. As compared to participants from the dental field, those from the medical field had 1.529 times higher odds to have good knowledge about pediatric OSA (95% confidence interval [CI]: 1.081–2.164), while those who belonged to institutions located in the central region had 1.83 times fewer odds to have good knowledge than those who belonged to institutions located in the southern region (95% CI: 0.337–0.886). Additionally, participants who belonged to public institutions had 3.45 times fewer odds to have good knowledge as compared to those who belonged to private institutions (95% CI: 0.176–0.477).

**Figure 1:**
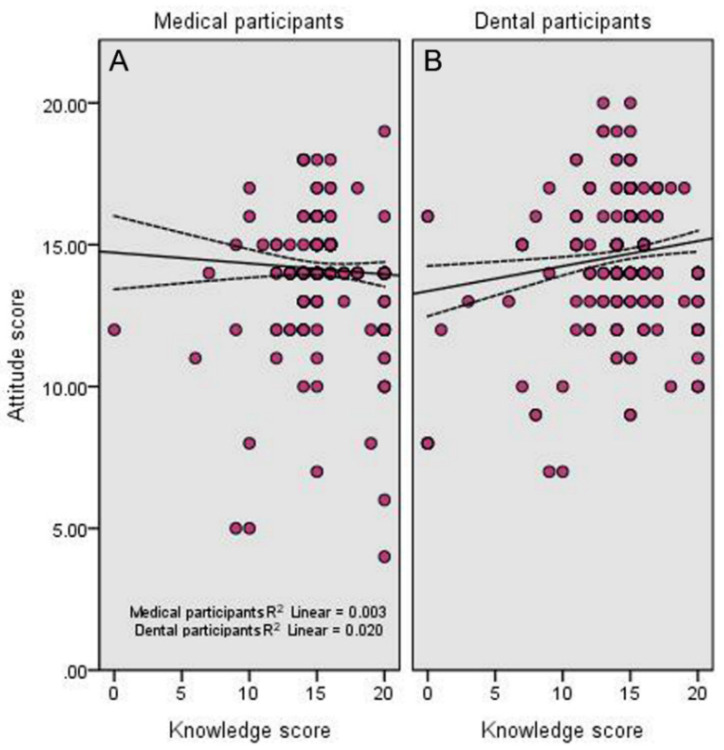
Relationship between the total attitude score and the total knowledge score of (**A**) medical participants and (**B**) dental participants. The middle lines show the line of best fit for the correlation and the two lateral lines show the 95% confidence intervals for the mean values.

Descriptive statistics of the participant’s responses to the questions which assessed their attitude about pediatric OSA are shown in Table 4. Altogether, 89.6% of the participants (91.2% of dental participants vs. 86.9% of medical participants) believed that pediatric OSA is an extremely important topic. Similarly, 93.3% of them (94.1% of dental participants vs. 92.3% of medical participants) considered identifying children with OSA as extremely important. The great majority of participants (80.3%), particularly from the dental field (81.4%), agreed or strongly agreed that they were confident in their ability to identify children at risk for OSA, as well as agreed or strongly agreed that they need further education about pediatric OSA (92.5% of total participants; 93.2% from the dental field). Finally, there was a significant positive correlation between the attitude score of dental participants and their knowledge score (*r* = 0.14, *p* = 0.004) (Figure 1). 

## 4. Discussion

This study is the first to assess knowledge of graduating medical and dental students as well as fresh graduates about pediatric OSA, particularly in Saudi Arabia. In the current study, the questionnaire which was used as the study instrument was developed to suit both medical and dental participants as OSAKA-KIDS, which was the only reliable questionnaire about pediatric OSA, focused on knowledge of physicians and was not designed for dentists or dental students [15,26,27]. Though a possibility still exists that the questionnaire we used did not cover all important information about pediatric OSA, it still contained some major topic areas which a fresh medical or dental graduate should know including the epidemiology, risk factors, diagnosis, signs and symptoms and whom to refer suspected cases. In-depth questions focusing on treatment were excluded as that is performed by otolaryngologists, sleep medicine specialists, or surgeons [3,7]. In the dental field, maxillofacial surgeons, orthodontists, and dentists specialized in oral medicine, dental sleep medicine or orofacial pain and dysfunction are those who may be involved with the treatment [14,28]; consequently, fresh graduates of both fields are expected to perform more patient screenings, initial diagnosis and referral of suspected cases than actual treatment. Nevertheless, it is essential to ensure that fresh graduates are aware of the effectiveness of intra-oral appliances in the management of pediatric OSA, which has been repeatedly emphasized, particularly in severe or complicated cases [3,12,29], when other treatment options do not resolve the condition (adenotonsillectomy) or are not tolerated (continuous positive airway pressure) [3,8].

Our findings revealed that knowledge of participants from both fields is very limited (30.5%) with a mean score of 14.95, despite good recognition of OSA as an important clinical disorder in children. This is concerning given the prevalent nature of pediatric OSA (3.4%), the high risk for sleep-disordered breathing (21%) in the kingdom [30], and that sleep in children is a major concern of parents [9]. A positive but weak correlation between knowledge and attitude scores of dental participants existed. Previously, Bian. [17] and Jokubauskas et al. [14] reported strong positive attitudes of dentists toward OSA despite having limited knowledge of the condition using different questionnaires, while Chang et al. [27] failed to find a strong positive correlation between physicians’ knowledge and attitudes about OSA in adults and children.

Similar studies which have used OSAKA-KIDS elsewhere reported higher knowledge levels of physicians about pediatric OSA (58.3–66.7%) [15,27] than that reported in the present study (30.5%). Many studies have reported lower knowledge levels of medical students and graduates about OSA than experienced doctors and specialists using the OSAKA questionnaire [15,21,22,23,24,25,27,31,32]. One of those studies reported that knowledge of physicians about pediatric OSA lagged behind that of adults [27]. This can be attributed to the lack of experience of students and fresh graduates [21,22]. Limited exposure to both didactic and clinical training about pediatric OSA in the undergraduate curricula is another important factor, particularly after considering that around half of the participants in this study (45.4%), regardless of the college type, were unaware if their curriculum included information about pediatric OSA. A great emphasis was placed on the underrepresentation of the topic of sleep medicine in the undergraduate medical curricula in general, including in Saudi Arabia [9,33,34,35]. Almohaya et al. [33] reported that during five years of medical education in Saudi medical schools, fewer than three hours are allocated to teaching sleep medicine, and in an assessment of Saudi medical students’ knowledge about sleep medicine, only 4.6% of the students showed good knowledge. It is also probable that pediatric OSA is not sufficiently covered in medical textbooks, which are a potential source of information for self-study [23]. Numerous obstacles to sleep medicine education in Saudi Arabia were identified including the lack of trained technicians, specialists, and funding issues [36]. 

A closer look at the individual questions in this study revealed that participants in both fields performed better in the questions about signs and symptoms of pediatric OSA. Similarly, Bian and Smith [1] and Vuorjoki-Ranti et al. [16] reported good knowledge of dentists about signs and symptoms of OSA using different questionnaires. This can partly explain the high level of confidence reported by participants from both fields in this study in identifying children at risk (80.3%). On the contrary, the questions about the epidemiology, risk factors, general findings (hyperactivity and learning deficits), and the possible need for intra-oral appliances in treatment, in the case of dental participants, were the most poorly performed. Compared with adults, children with OSA can present with hyperactivity, emotional difficulties, decreased academic performance, and difficulty in concentration [37]. Consequently, it is crucial to enrich the undergraduate medical and dental curricula with these learning objectives.

Of all sociodemographic variables in the current study, the college type (medicine), institution sector (private), as well as institution’s location in the kingdom (southern) were predictors of good knowledge according to the multivariate analysis; therefore, both null hypotheses tested were rejected. The greater familiarity of medical participants with pediatric OSA is perhaps due to different training between medical and dental Saudi schools. Saudi medical schools likely provide better exposure of the students to the diagnosis and perhaps treatment of pediatric OSA during their clerkships as compared to dental schools. The differences in clinical training between different Saudi institutions and the public versus private institutions may also explain the higher knowledge levels observed among institutions according to their location and sector. It is also likely that the participants who had good knowledge acquired their knowledge through self-study [38]. 

There are some limitations of the present study which warrant caution in the interpretation of the results; first, we have used a convenience sample of medical and dental students and graduates from Saudi Arabia. Consequently, we are not claiming our study to be representative or provide an accurate reflection of the knowledge levels, despite the fact that the participants belonged to institutions from both sectors and all five geographical regions of Saudi Arabia. Additionally, we cannot generalize our results to other countries where medical or dental education about the topic might differ in important ways. Another limitation would be that this was a cross-sectional study, and we cannot infer causation from any of the associations we observed. Nevertheless, the present study still provides baseline data which warrant further investigation in future studies.

## 5. Conclusions

The knowledge level of graduating Saudi medical and dental students and fresh graduates about pediatric OSA was not optimal. The college type, institution sector, and location in the kingdom were factors associated with good knowledge. This study highlights the need to incorporate effective pediatric OSA education into undergraduate and continuous medical and dental education programs in Saudi Arabia (CME) after graduation. This should ensure early diagnosis of children at risk, appropriate referral and treatment provision, and reduced morbidity and risk of complications.

## Figures and Tables

**Table 1:** Frequency (percentage) of the correct answer in general knowledge questions about pediatric OSA.

Question	Medical N = 297	Dental N = 425	Total N = 722	*p*-Value
Children with OSA may present with hyperactivity *Correct response: True*	67 (22.6)	65 (15.3)	132 (18.3)	0.009 *
A polysomnogram is needed to diagnose obstructive sleep apnea in children *Correct response: True*	270 (90.9)	376 (88.2)	646 (89.5)	0.177
Enlarged tonsils and adenoids, as well as obesity, are considered risk factors for OSA *Correct response: True*	288 (97)	409 (96.2)	697 (96.5)	0.377
Children with suspected OSA should have a thorough head and neck and oropharyngeal examination *Correct response: True*	293 (98.7)	406 (95.5)	699 (96.8)	0.013 *
Children with OSA may have learning deficits *Correct response: True*	122 (41.1)	103 (24.3)	225 (31.2)	<0.0001 *
Referral to an otolaryngologist, pulmonologist, or sleep medicine physician and a dental sleep medicine specialist (e.g., orthodontist) is necessary for any child suspected of having OSA *Correct response: True*	287 (96.6)	403 (94.8)	690 (95.7)	0.2
Treatment of OSA in children can include intra-oral appliances *Correct response: True*	201 (67.7)	196 (46.1)	397 (55)	<0.0001 *
A common cause of OSA in children is congestive heart failure *Correct response: False*	110 (37)	239 (56.2)	349 (48.3)	<0.0001 *
Nearly 2% of children have OSA *Correct response: True*	70 (23.6)	91 (21.4)	161 (22.3)	0.276
OSA occurs most often in children older than 8 years of age *Correct response: False*	9 (3.3)	14 (3.3)	23 (3.2)	0.314

The correct response in italics, OSA: obstructive sleep apnea, * indicates a statistically significant difference.

**Table 2:** Frequency (percentage) of the correct answer in questions about signs and symptoms of pediatric OSA.

Question	Medical N = 297	Dental N = 425	Total N = 722	*p*-Value
Excessive daytime sleepiness and restlessness *Correct response: True*	289 (97.3)	394 (92.7)	683 (94.6)	0.005 *
Loud snoring three or more nights per week *Correct response: True*	291 (98)	402 (94.6)	693 (96)	0.016 *
Episodes of breathing cessation witnessed by another person *Correct response: True*	291 (98)	401 (94.4)	692 (95.8)	0.011 *
Abrupt awakenings accompanied by shortness of breath *Correct response: True*	291 (98)	401 (94.4)	692 (95.8)	0.011 *
Awakening with a dry mouth or sore throat *Correct response: True*	287 (96.6)	397 (93.4)	684 (94.7)	0.039 *
Morning headache *Correct response: True*	275 (92.6)	376 (88.5)	651 (90.2)	0.043 *
Difficulty staying asleep *Correct response: True*	269 (90.6)	388 (91.5)	657 (91.1)	0.379
Attention problems *Correct response: True*	165 (55.7)	188 (44.2)	353 (49)	0.002 *
Mouth breathing *Correct response: True*	289 (97.3)	405 (95.3)	694 (96.1)	0.117
Sweating *Correct response: True*	83 (27.9)	79 (18.6)	162 (22.4)	0.002 *
Bedwetting *Correct response: True*	60 (20.2)	73 (17.2)	133 (18.4)	0.175
Waking up a lot *Correct response: True*	285 (96)	395 (92.9)	680 (94.2)	0.059

The correct response in italics, OSA: obstructive sleep apnea, * indicates a statistically significant difference.

**Table 3:** The sociodemographic background of the participants and its relation with good knowledge about pediatric OSA.

Question	Categories	N (%)	N (%) with Good Knowledge	*p*-Value ^†^	B	Odds Ratio	95% CI	*p*-Value ^‡^
Gender	Male	397 (54.99)	113 (28.5)	0.112	Not entered in the regression model			
	Female ^reference^	325 (45.01)	107 (32.9)					
Age group in years	18–22	427 (59.1)	135 (31.6)	0.235	Not entered in the regression model			
	23–27 ^reference^	295 (40.9)	85 (28.8)					
College	Medicine	297 (41.1)	113 (38.0)	<0.0001 *	0.425	1.529	1.081–2.164	0.016 *
	Dentistry ^reference^	425 (58.9)	107 (25.2)					
Academic level	Fifth-year student	442 (61.2)	132 (29.9)	0.679	Not entered in the regression model			
	Intern ^reference^	280 (38.8)	88 (31.4)					
Institution location in the kingdom	Central	287 (39.8)	65 (22.6)	0.005 *	−0.604	0.546	0.337–0.886	0.014 *
	Eastern	92 (12.7)	29 (31.5)		−0.486	0.615	0.328–1.152	0.129
	Western	133 (18.4)	50 (37.6)		−0.120	0.887	0.523–1.504	0.657
	Northern	94 (13.0)	33 (35.1)		−0.085	0.918	0.510–1.654	0.776
	Southern ^reference^	116 (16.1)	43 (37.1)					
Institution sector	Public	643 (89.1)	175 (27.2)	<0.0001 *	−1.238	0.290	0.176–0.477	<0.0001 *
	Private ^reference^	79 (10.9)	45 (57)					
The curriculum included information about OSA in children	Yes	466 (54.5)	159 (34.1)	0.010 *	0.274	1.316	0.888–1.950	0.172
	No	31 (10.9)	5 (16.1)		−0.291	0.747	0.262–2.134	0.586
	Do not know ^reference^	225 (31.2)	56 (24.9)					

* indicates a statistically significant difference, ^†^ using chi-square test and ^‡^ binary logistic regression analysis, OSA: obstructive sleep apnea, CI: confidence interval.

**Table 4:** Mean and SD of participants’ responses in attitude questions about pediatric OSA.

Question	Medical	Dental	Total
As a clinical disorder, OSA is *	3.10 ± 0.702	3.33 ± 0.733	3.24 ± 0.728
Identifying children with possible OSA is *	3.29 ± 0.723	3.33 ± 0.701	3.31 ± 0.710
I feel confident identifying children at risk for OSA †	3.80 ± 0.684	3.92 ± 0.923	3.88 ± 0.835
I need further education about OSA †	3.94 ± 0.551	4.07 ± 0.603	4.01 ± 0.585

OSA; obstructive sleep apnea. * response categories: not important = 1, somewhat important = 2, important = 3, very important = 4, extremely important = 5. † response categories: strongly disagree = 1, disagree = 2, neither agree nor disagree = 3, agree = 4, strongly agree = 5.

## Data Availability

The data used to support the findings of this study can be made available upon request to the corresponding author.
